# Awareness of female students attending higher educational institutions toward legalization of safe abortion and associated factors, Harari Region, Eastern Ethiopia: a cross sectional study

**DOI:** 10.1186/s12978-015-0006-y

**Published:** 2015-03-17

**Authors:** Ayele Geleto, Jote Markos

**Affiliations:** Department of Public Health, College of Health and Medical Science, Haramaya University, Haramaya, Ethiopia; School of Nursing and Midwifery, College of Health and Medical Science, Haramaya University, Haramaya, Ethiopia

**Keywords:** Safe abortion, Legalization, Awareness, Penal code, Female college student

## Abstract

**Background:**

Unsafe abortion has been recognized as an important public health problem in the world. It accounts for 14% of all maternal deaths in sub-Saharan African countries. In Ethiopia, 32% of all maternal deaths are accounted to unsafe abortion. Taking the problem of unsafe abortion into consideration, the penal code of Ethiopia was amended in 2005, to permit safe abortion under a set of circumstances. However, lack of awareness on the revised penal code is a major barrier that hinders women to seek safe abortion. The aim of this study is to assess awareness of female students attending higher educational institutions toward legalization of safe abortion and associated factors in Harari region, eastern Ethiopia.

**Methods:**

Institution-based descriptive cross sectional study was conducted among 762 female students who are attending five higher educational institutions in Harari Region. Systematic sampling method was used to identify study participants from randomly selected colleges. Self administered structured questionnaire was used to collect data. Data were entered in to Epi Info version 6.04 and analyzed by SPSS version 17.0 statistical packages. Frequency, percentage and ratio were used to describe variables. Multivariable logistic regression analysis was done to control confounders and odds ratio with 95% confidence interval was used to identify factors associated with awareness of female students to legalization of abortion.

**Results:**

762 study participants completed the survey questionnaire making the response rate 90.2%. Only 272 (35.7%) of the respondents reported that they have good awareness about legalization of safe abortion. Studying other fields than health and medicine [AOR 0.48; 95%CI (0.23, 0.85)], being the only child for their family [AOR 0.28; 95%CI (0.13, 0.86)], having no boy friend [AOR 0.34; 95%CI (0.12, 0.74)], using family planning [AOR 0.50; 95%CI (0.13 and 0.86)], being 25 years or older [AOR 1.64; 95%CI (1.33, 2.80)] were significantly associated with awareness of female students to legalization of safe abortion.

**Conclusions:**

Only slightly more than a third of the study participants, 35.7% have good awareness of legalization of safe abortion. Strengthening information dissemination regarding legalization of safe abortion is required for female reproductive age group in general and higher institution female students in particular.

## Introduction

Abortion is the termination of pregnancy by removal or expulsion of a conception tissue (fetus, fetal membranes and placenta) from the uterus. Abortion can occur either spontaneously, due to complications during pregnancy or could be induced [1]. Safe abortion can be defined as providing services for termination of a viable early pregnancy as well as managing other clinical types of abortion. The World Health Organization (WHO) defines unsafe abortion as a procedure for terminating unwanted pregnancy performed by persons lacking the necessary skills or in an environment not in conformity with minimal medical standards, or both [2].

Unsafe abortion is a significant cause of maternal mortality and morbidity in the world. 95% of unsafe abortion takes place in developing countries. Globally, each year, unsafe abortion claims the lives of about 68,000 women; 43% of these women were from Africa. Unsafe abortion accounts for an estimated 14% of maternal death in Africa [2] and 32% in Ethiopia [3]. Despite the global effort to improve post abortion care, rising contraceptive use and easing abortion restrictions, unsafe abortion continues to be common in Africa [4,5]. In Ethiopian society as premarital sex is taboo, unmarried adolescents are discouraged from using any kind of contraceptives. This socio-cultural barrier to use contraceptives could result in unwanted teenage pregnancy. Lack of awareness to legalization of safe abortion forces unmarried adolescents to seek unsafe abortion in a secret place [6].

In Ethiopia, before 2005, abortion was permitted only to save the life of a pregnant woman. The woman seeking abortion service needs to be diagnosed by an obstetrician/gynecologist to have a life threat grave danger. In 2005, the penal code was amended to permit abortion under a much broader set of circumstances. These amendments include i) if the pregnancy is a result of rape or incest, ii) if continuation of the pregnancy endangers the life of the mother, iii) if the fetus has an incurable and serious deformity, iv) if the pregnant woman, owing to a physical or mental deficiency she suffers from, or she is physically as well as mentally unfit to bring up the child, v) in the case of life threat grave and imminent danger which can be averted only by an immediate intervention, an act of terminating pregnancy is not punishable [7].

In most of developing countries including Ethiopia, access to safe abortion continues to depend on women’s awareness of the legal background of safe abortion. Although the new 2005 Ethiopian abortion law is relatively liberal, due to lack of knowledge of legal rights among most women, shortage of safe abortion services provision and significant amount of socio-cultural pressures, women still seek unsafe abortion services [8]. In Ethiopia, currently both medical abortion (Mifepristone/Misoprostol) and surgical abortion (MVA) are used to safely end a pregnancy [9]. But limited awareness on the revised 2005 penal code of the Federal Democratic Republic of Ethiopia (FDRE) is one of the major obstacles that hinder women from obtaining Comprehensive Abortion Care (CAC). Therefore, the main aim of this research is to assess the level of awareness of female students learning at higher educational institutions to legalized safe abortion in Harari regional state, eastern Ethiopia.

## Methods

### Study Design and setting

Institution-based quantitative cross-sectional study design was conducted among 762 female students attending higher educational institutions in Harari regional state. Harar is the capital city of Harari region, and is 526 km far from Addis Ababa, the capital city of Ethiopia, to the east. The region has nine woreda administration structures. Three of the weredas are rural and six are urban. The urban woredas are sub- divided in to nineteen kebeles, whereas the rural woredas are sub divided in to seventeen peasant associations. The total population of the region was 205,000 from which 54.8% were urban dwellers. Females in reproductive age group were 43,050. There are three public and five private higher educational institutions in Harari Region. Public colleges include College of Health and Medical Science of Haramaya University, Harar Teacher’s Training College and Harar College of Health Sciences. The private higher educational institutions are Rift Valley University College, Afran Qallo College, Lucy College, Horn international College and East Africa College of health sciences. The study was conducted from January to March 2012 among female students attending two public and three private higher educational institutions found in the region.

### Study participants

Randomly selected day time female students attending higher educational institutions in Harari Region were included in the study. The data were collected from voluntary female college students. Students with mental problem and seriously ill who cannot provide appropriate information were excluded from the study.

### Sampling method

To calculate the sample size a single population proportion formula, [n = (Z α/2)^2^ p (1-p) / d2], was used. Since the proportion of awareness of female students to legalization of safe abortion in Ethiopia is not yet known, P = 50% was used to obtain maximum sample size. In addition, 95% confidence level 5% margin of error (d = 0.05), design effect of 2 and non-response rate of 10% were considered. Therefore, the final sample size was calculated to be 845. To collect sample from each college, the colleges were stratified into governmental and private higher educational institutions. Then two governmental educational institutions (College of health and Medical Science of Haramaya University and Harar Teacher’s Training College) and three private colleges (Rift Valley University College, Afran Qallo College and Horn International College) were randomly selected to be included in the study. The sample size was proportionally allocated to all the departments based on the number of female students in each department. Female students from all study years were included. The study participants were identified from all departments of selected colleges by systematic sampling method from the list of female students found in each department.

### Data collection

A self administered pre tested structured questionnaire was used for data collection. Close ended questionnaire was developed in English language and translated to local languages: *Amharic and Afaan Oromoo* for data collection*.* After completion of data collection the questionnaire was retranslated to English language for analysis. These questionnaires were developed after completion of a literature review and pretested on 40 female college students (5% of sample size) attending colleges other than the sampled colleges. Feedback obtained from pretest was incorporated and the survey tool was revised and finalized after pretest. Data collectors were selected from health professionals those teaching at College of Health and Medical Science, Haramaya University**.** They were given training before data collection was commenced. Completed questionnaires were checked every day by investigators and supervisors.

### Data analysis

Data were entered into Epi Info Version 6.04 and were analyzed by SPSS version 17 statistical packages for window. Descriptive statistics was used to summarize the data and the results were presented using frequency tables and percentages. A multivariate logistic regression analysis was employed to control confounders between variables. Crude Odds ratio with 95%CI was used to determine presence of association between explanatory variables and level of awareness of respondents to legalization of safe abortion. The degree of association between dependent and independent variables was measured using adjusted odds ratio with 95% confidence interval at significance level of ≤ 0.05.

### Measurements

Dependent variable for this study is level of awareness of female students attending higher educational institutions toward legalization of safe abortion. To measure level of awareness, the mean values of the respondents to the five criteria under which safe abortion is legally allowed was calculated and taken as a cut point value to determine whether female students have good or poor awareness about legalization of safe abortion. Thus, female students for whom score was below mean (490 female students) were considered as having poor awareness and those with mean and above score (272 female students) were regarded as having good awareness. Socio-demographic variables including participants’ age, marital status, religion, year of study, department of study, and previous sexual exposure were independent variables. Female students who had boy friend and whether they were learning colleges were also independent variables in the study.

### Data quality control

The questionnaire was pre-tested and feedback was used to make modifications to the questionnaires. Members of field staff (data collectors and supervisors) were selected according to their qualifications, work experience in the field of data collection and experience in carrying out surveys. They were given extensive training before data collection was commenced. During training, the objective of the study, method of data collection and supervision were discussed. Furthermore, each question included in the questionnaire was discussed in detail. Field practice (pre-test) was undertaken to check the practicality and applicability of the questionnaire. Each day, collected data were checked for its completeness and consistence by supervisors and principal investigator. Data were also cleaned and rechecked after double data entry was performed.

### Ethical consideration

The study was approved by Institutional Research Ethics Review Committee (IRERC) of College of Health and Medical Science, Haramaya University. Official letters of co-operation were written to all participant higher educational institutions by College of Health and Medical Science, Haramaya University. A letter explaining about the purpose, method and anticipated benefit and risk of the study was attached to each questionnaire. It was explained for the respondents that participation in this study was voluntary and private information would be protected. Written informed consent was obtained from each participant. In order to protect confidentiality, participants’ names and ID numbers were not included in the questionnaire.

## Result

### Socio-demographic characteristics of the respondents

A total of 762 respondents completed the survey questionnaires from the proposed 845 study participants, making response rate of 90.2%. Majority of the respondents (72%) were aged between 20–25 years. Amhara ethnic group accounted for nearly half of the study participants (47.2%). Majority of the respondents, 71.1% were Christian by religion and about four fifth of the respondents were single. More than two fifth, 43.2% of the respondents were middle child in their birth order while 2.8% were the only child to their family. For about 76.8% of the respondents, their father and mother are alive and only 1.7% of them have lost both of their parents (Table 1).

**Table 1:** **Socio-demographic characteristics of respondents (n = 762), Harari Region, Eastern Ethiopia, 2012**

**Characteristics**	**Frequency**	**Percent**
**Age**		
**25 years and less**	682	89.5
**More than 25 years**	80	10.5
**Ethnicity**		
Oromo	272	35.7
Amhara	360	47.2
Others	130	17.1
**Religion**		
Christian	542	71.1
Muslim	220	28.9
**Marital status**		
Single	620	81.4
Married	142	18.6
**Resident of respondent's family**		
Urban	606	79.4
Rural	156	20.6
**Respondent's resident**		
In the campus	279	36.6
Out of the campus	483	63.4
**Department of the respondent**		
Health and medicine	563	73.8
Other field of study	199	26.2
**Year of study of the respondents**		
Two years and less	319	41.8
More than two years	443	58.2
**Birth order**		
First	270	35.4
Middle	329	43.2
Last	142	18.6
The only child	21	2.8
**Life status of respondents’ family**		
Both are alive	585	76.8
Only mother alive	146	19.2
Only father alive	18	2.4
Both are died	13	1.7

### Reproductive health of the respondents

Nearly two fifth, (42.9%) of the respondents in the study had a boyfriend and 184 (24.1%) of the study participants responded that they had ever performed sexual intercourse. Among those who had sexual intercourse, 17% of them had sex before the age of 15 years. And majority of them 75.3% had sex by the age of 18 years. 533 (69.9%) of the study respondents knew about emergency contraception. Only 68 study participants had a pregnancy from which only three were pregnant at the time of data collection. Forty seven of the pregnancies resulted in a live birth while eighteen were terminated by abortion. Twelve of the abortions were unsafe (induced by traditional practitioner) and only six abortions had been conducted in health institutions, both public and private, where it was assumed to be conducted under safe condition by trained health professionals. To induce abortion, five of the respondents reported that they were given traditional leafs and roots to drink. Among study participants for whom abortion was performed, seven of them were provided unknown tablet to swallow to induce abortion. For six of them, traditional practitioners inserted plastic material into their cervix to induce abortion. Twenty nine of the respondents reported that they had rape history and nine of them had got pregnancy as a result of rape victim (Table 2).

**Table 2:** **Reproductive health of the respondents, Harari Region, Eastern Ethiopia 2012**

**Characteristics**	**Frequency**	**Percent**
**Have boy friend (n = 762)**		
Yes	327	42.9
No	435	57.1
**Ever had sexual intercourse (n = 762)**		
Yes	184	24.1
No	578	75.9
**Age at first sexual intercourse (n = 184)**		
<15	32	17.4
15-20	137	74.4
>20	15	8.2
**Family planning use (n = 184)**		
Yes	152	82.6
No	32	17.4
**Type of family planning used (n = 152)**		
Artificial contraceptives	138	90.7
Natural family planning methods	14	9.3
**Knowledge of emergency contraceptive (n = 762)**		
Yes	533	69.9
No	229	30.1
**Have ever got pregnant (n = 184)**		
Yes	68	36.9
No	116	63.1
**Outcome of the pregnancy (n = 68)**		
Aborted	18	26.5
Delivered	47	69.1
Know pregnant	3	4.4
**History of rape victim (n = 762)**		
Yes	29	3.8
No	733	96.2
**Result of rape victim (n = 29)**		
Got pregnant	9	31
Develop genital swelling	2	6.9
Develop genital ulcer	3	10.3
Develop vaginal discharge	15	51.8

**Figure 1:**
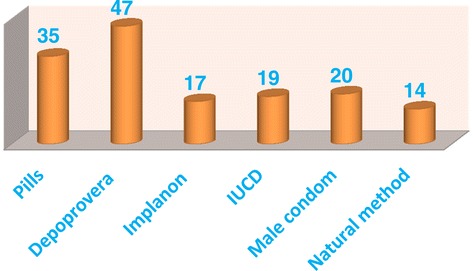
**Types of family planning methods utilized by participants, Harari Region, Eastern Ethiopia 2012.**

Amongst the adolescents who reported to had sex, 82.6% used a contraceptive method to prevent pregnancy. About 30.9% of them used Depo-Provera while only 9.2% of the respondents used natural family planning method. (Figure 1) From 138 artificial family planning users 64 (46.4%) of them got the method from public health institutions. Twenty six percent got the method from private pharmacies while 23.2% and 4.4% get family planning methods from private clinics and shops respectively.

### Awareness of respondents about legalization of safe abortion

**Figure 2:**
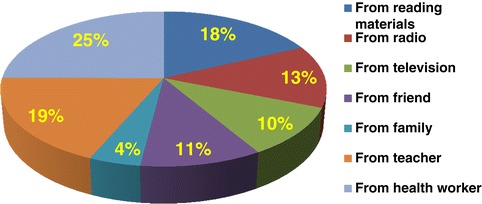
**Sources of information about the criteria under which safe abortion is legally allowed among female students of higher educational institutions, Harari region, eastern Ethiopia, 2012.**

Only 272 (35.7%) of the respondents have good awareness about legal background of safe abortion. Nearly 13% of the study participants responded that safe abortion is legally allowed for all types of pregnancy. Slightly more than half, 52% of the respondents reported that safe abortion is legally allowed if the pregnancy is the result of incest and 49.1% responded safe abortion is legally allowed if the pregnancy is the result of rape. Four hundred thirteen (54.2%) reported that safe abortion is legally allowed if the continuation of the pregnancy endangers the life of the mother or the child or the health of the mother or where the birth of the child is a risk to the life or health of the mother. Only 27% had awareness that safe abortion is legally allowed if the fetus has an incurable and serious deformity. About a fifth of study participants responded that safe abortion is legally allowed if the pregnant woman, owing to a physical or mental deficiency she suffers from, or if she is physically as well as mentally unfit to bring up the child (Table 3). Twenty five percent of the respondents have received information about criteria, under which abortion is legally allowed, from health workers while only 4% reported their family as a source of information (Figure 2).

**Table 3:** **Level of awareness of the respondents to the criteria under which safe abortion is legally allowed in Ethiopia, Harari Region, Eastern Ethiopia 2012**

**Characteristics**	**Frequency**	**Percent**
**Safe abortion is legally allowed for all type of pregnancy**		
Yes	97	12.7
No	665	87.3
**Safe abortion is allowed if pregnancy is the result of incest**		
Yes	396	52.0
No	366	48.0
**Safe abortion is allowed if pregnancy is the result of rape**		
Yes	374	49.1
No	388	50.9
**Safe abortion is allowed if the continuation of the pregnancy endangers the life of the mother or the child**		
Yes	413	54.2
No	349	45.8
**Safe abortion is allowed if the fetus has an incurable and serious deformity**		
Yes	206	27.0
No	556	73.0
**Safe abortion is allowed if the pregnant woman has physical or mental deficiency or she is physically and mentally unfit to bring up the child**		
Ye	150	19.7
No	612	80.3
**Average**		
Yes	272	35.7
No	490	64.3

### Factors associated with awareness of legalization of safe abortion

Multivariate logistic regression analysis showed that age, birth order, having boy friend, field of study and years of study were some of the factors that were significantly associated with awareness of female students to legalization of safe abortion after possible confounders were controlled. Female students learning another field of study than health and medical science [AOR 0.48; 95%CI (0.23, 0.85)] and those who were the only child for their family than the first child [AOR 0.28; 95%CI (0.13, 0.86)] were less likely to have good awareness about legalization of safe abortion. Those who had no boyfriends [AOR 0.34; 95%CI (0.12, 0.74)] and who used family planning method during sexual intercourse [AOR 0.50; 95%CI (0.13, 0.86)] were less likely to have good awareness about legalization of safe abortion. Those female students who were aged 25 years or above were 1.6 times more likely to have good awareness about legalization of safe abortion with the reference to younger than 25 years, [AOR 1.64; 95%CI(1.33,2.80)]. Married students [AOR 1.82; 95%CI91.12, 3.52)], those studied for more than two years [AOR 2.34; 95%CI (1.18, 5.97)] and those who had lost both of their parents [AOR 1.70; 95%CI (91.23, 5.66)] were more likely to have good awareness about legalization of safe abortion than single female students, those studied for two years or less and whose both families were alive (Table 4).

**Table 4:** **Association of respondents’ characteristics with level of awareness to legalization of safe abortion, Harari Region, Eastern Ethiopia 2012**

**Characteristics**	**Awareness to legal abortion**		**COR (95%CI)**	**AOR (95%CI)**
	**Good**	**Poor**		
**Age**	N (%)	N (%)		
25 years and less	252	430	1.00	1.00
More than 25 years	20	60	1.75 (1.25,2.61)*	1.64 (1.33,2.80)*
**Ethnicity**				
Oromo	91	181	1.00	1.00
Amhara	124	236	0.95 (0.66,1.92)	0.42 (0.12,1.81)
Others	57	73	0.64 (0.31,1.12)	0.67 (0.22,0.99)
**Religion**				
Christian	219	323	1.00	1.00
Muslim	53	167	2.13 (1.29,4.58)*	1.46 (0.98,5.80)
**Marital status**				
Single	225	395	1.00	1.00
Married	47	95	1.15 (1.04,3.21)*	1.82(1.12,3.52)*
**Resident of respondent's family**				
Urban	172	434	1.00	1.00
Rural	100	56	0.22 (0.15,0.68)*	0.53 (0.11,1.10)
**Respondent's resident**				
In the campus	137	142	1.00	1.00
Out of the campus	135	348	2.48 (1.28,4.77)*	1.88(0.73,3.42)
**Department of the respondent**				
Health and medicine	137	426	1.00	1.00
Other field of study	135	64	0.15 (0.10,0.73)*	0.48 (0.23,0.85)*
**Year of study of the respondents**				
Two years and less	121	198	1.00	1.00
More than two years	151	292	1.18 (0.98,2.91)	2.34 (1.18,5.97)*
**Birth order**				
First	99	171	1.00	1.00
Middle	139	190	0.79 (0.52,1.73)	0.43 (0.11,1.23)
Last	18	124	3.98 (1.62,8.10)*	1.18 (0.99,6.21)
The only child	16	5	0.18 (0.10,0.88)*	0.28 (0.13,0.86)*
**Life status of respondents’ family**				
Both are alive	212	373	1.00	1.00
Only mother alive	48	98	1.16 (0.87,3.97)	1.19 (0.78,5.44)
Only father alive	8	10	0.71 (0.75,2.31)	0.79 (0.56,2.65)
Both are died	4	9	1.27 (1.04,3.84)*	1.7 (1.23,5.66)*
**Have boy friend (n = 762)**				
Yes	101	226	1.00	1.00
No	171	264	0.68 (0.14,0.89)*	0.34 (0.12,0.74)*
**Ever had sexual intercourse (n = 762)**				
Yes	61	123	1.00	1.00
No	211	367	0.86 (0.23,1.02)	1.83 (0.76,3.57)
**Family planning use (n = 184)**				
Yes	38	114	1.00	1.00
No	234	376	0.53 (0.13,0.82)*	0.50 (0.13,0.86)*
**Have ever got pregnant (n = 184)**				
Yes	26	42	1.00	1.00
No	246	448	1.12 (0.59,2.01)	0.36 (0.19,0.86)*

*= p ≤ 0.05.

## Discussion

The findings of our study have shown that fewer than half, 35.7% of the respondents, had good awareness about legal background of safe abortion in Ethiopia. A similar finding was reported in the study conducted in Nepal. The baseline survey conducted in Nepal in 2003 showed that only 15% of the 1,100 rural married women of reproductive age (MWRA) interviewed were aware of the new abortion law, and 56 percent still believed that abortion was illegal in the country [10]. However, a study conducted in Latin America found over 60 percent of all women surveyed was aware of medications to induce abortion [11]. It is also consistent with the study findings in South Africa where only 264(32%) of 831 study participants did not know that the law in South Africa allowed for legal abortion [3]. Similar finding was reported from study conducted in Ethiopia where 552(67.9%) of college students have awareness of legal abortion [12]. Lower awareness of students toward legalization of safe abortion in Ethiopia could be due to poor information dissemination to the target population and poor information seeking of adolescents about their reproductive health as compared to the case of developed country. Therefore, policy alone does only have a limited effect on the health and lives of women. Accessing to safe abortion service and improving women’s awareness are crucial to reduce mortality and morbidity related to unsafe abortion.

Abortion has been legalized in Ethiopia since 2005 under some circumstances. But 67% of the abortions performed in our study were unsafe (performed by traditional method outside of health institutions). The result of this study is consistent with the findings in India where nearly 40 years after India legalized abortion Indian women continue to be unaware that safe abortion service was available or were unable to access it. Although abortion has been legal in India for decades, unsafe abortions were estimated to be 90 percent [8]. Our findings were also similar to the case of Africa where about 5.5 million African women undergo unsafe abortions each year. East Africa in particular has one of the world’s highest rates of maternal deaths linked to complications from unsafe abortions. Over 50% of all women seeking abortions in Ethiopia do so outside the reach of trained medical professionals and outside of health facilities even after legalization of safe abortion services [5]. Nearly two-third of the abortions in our study was unsafe. This might be due to stigma and the erroneous belief of the community toward abortion which enforces adolescent women to choose secrecy over safety.

The findings of our study indicated that, to induce abortion traditional methods were used in majority of the case. Among the study participants for whom abortion was performed, five of them were given leaves and roots to drink. An unknown tablet was given to swallow for seven of them and plastic material was inserted into their cervix for six to induce abortion. This finding was consistent with the result of the study conducted in northwest Ethiopia where plastic tube was inserted in to the cervix of 54.7% participants and different oral drugs were given for 35.9% to induce the abortion [8]. The possible reason for using such traditional method of inducing abortion is that most of the traditional practitioners have no training on medical profession. They didn’t understand the health adverse effect of using such leaves, roots and plastic tubes.

In our study, age of respondents, birth order*,* place of residence, contraceptive use, and type of education, years of study were some of the factors that were significantly associated with awareness of female students to legalization of safe abortion. Similar factors were reported in a study done in northwestern Ethiopia where place of residence, marital status, contraceptive use, number of pregnancies and level of education attained by the women were reported as determinant factors of unsafe abortion [8]. Another study conducted in Ethiopia also reported that field of study and having had sexual intercourse are associated to attitude of female students toward legalization of safe abortion [12].

### Limitation of the study

The study suffered from the usual limitation of a cross sectional study. The study also did not discover information about uneducated adolescents living both in urban and rural areas of the region. So the finding of this study could only be generalized for educated adolescents in the region. Moreover, the nature of the sensitivity of the abortion might result in information bias and past history on abortion might be affected by recall bias. Limited numbers of similar studies were conducted in Ethiopia to compare our findings with.

## Conclusion

The study disclosed that there was high proportion of female college students with poor knowledge of legalization of safe abortion in the study area. Moreover, this poor knowledge urges women to practice unsafe abortion which in turn leads to maternal mortality and morbidity. Much more effort should be done on information education and communication of awareness creation on legal background of safe abortion. Furthermore, researches should be conducted to assess the awareness of rural and less educated adolescents toward legalization of safe abortion. Studies exploring the attitude-practice gap should be done on women of abortion and early initiation of sexual intercourse to see the cause and effect relationship clearly.

## Abbreviations

AOR: Adjusted Odds Ratio
CAC: Comprehensive Abortion Care
COR: Crude Odds Ratio
EDHS: Ethiopian Demographic and Health Survey
FDRE: Federal Democratic Republic of Ethiopia
IRERC: Institutional Research and Ethical Review Committee
MVA: Manual Vacuum Aspiration
WHO: World Health Organization
